# Exploring compassionate care in rehabilitation among individuals who are involved with the criminal-legal system with traumatic brain injury: A scoping review

**DOI:** 10.1371/journal.pone.0341381

**Published:** 2026-06-24

**Authors:** Martina Rubino, Caelin Mason, Giacomo Campo, Vincy Chan

**Affiliations:** 1 Institute of Forensic Sciences, University of Toronto, Mississauga, Ontario, Canada; 2 Department of Biology, University of Toronto, Mississauga, Ontario, Canada; 3 KITE Research Institute, Toronto Rehabilitation Institute-University Health Network, Toronto, Ontario, Canada; 4 Institute of Health Policy, Management and Evaluation, University of Toronto, Toronto, Ontario, Canada; 5 Rehabilitation Sciences Institute, University of Toronto, Toronto, Ontario, Canada; Royal Infirmary of Edinburgh, UNITED KINGDOM OF GREAT BRITAIN AND NORTHERN IRELAND

## Abstract

Traumatic brain injury (TBI) is disproportionately prevalent among individuals who are involved with the criminal-legal system and can impact one’s ability to critically think and emotionally regulate, thereby increasing risk of aggressive behaviour and recidivism. Considering and providing compassionate care can improve healthcare experiences and outcomes for this population. This scoping review aimed to answer the question: “To what extent is compassionate care considered or provided in rehabilitation for individuals who are involved with the criminal-legal system with TBI?” A systematic search through MEDLINE, Embase, APA PsycINFO, Cochrane Central Register, Nursing and Allied Health Premium, Criminal Justice Abstracts, CINAHL Complete, and Applied Social Sciences Index & Abstracts was established, and articles that met predetermined inclusion criteria were identified. A descriptive numerical summary and qualitative analytical techniques were applied to analyse the data. Twenty-five articles met the inclusion criteria and 24% (n = 6) of included articles were identified to provide/consider compassionate care through awareness of suffering, judgement and consideration, and motivation to alleviate suffering in rehabilitation programs, interventions, or services provided by healthcare providers or professional disciplines. While findings indicate dominance of Western contexts and a lack of routine consideration and/or provision of compassionate care, it highlights the following opportunities to integrate compassionate care in rehabilitation programs or services for those who intersect the criminal-legal system with TBI: (1) routine TBI and criminal-legal system-related education and TBI screening to increase awareness of patient suffering, (2) person-centred frameworks to address judgement and consideration of patients’ suffering, and (3) establish relationships between patients and service providers and monitor progress throughout rehabilitation to alleviate patient suffering. Research on the role of compassionate care in rehabilitation outcomes, how compassionate care may be routinely integrated, and its impact (positive or negative) on individuals are encouraged. Research globally is also encouraged, as many of the included articles/studies in this review were published in Western countries, with most of them originating from the United States. As such, findings from this review may represent a Western perspective on compassionate care and may not be generalizable to other countries.

## Introduction

Traumatic brain injury (TBI) is one of the leading causes of disabilities worldwide [1]. Menon and colleagues defined TBI as “an alteration in brain function or other evidence of brain pathology caused by an external force” [2]. These alterations can result in chronic detrimental effects represented as reduced abilities in essential cognitive processes (i.e., problem-solving skills, memory, executive and motor functioning) [1,3,4]. This can then impact an individual’s ability to regulate emotions, effectively opening the door for increased risk-taking behaviours [5]. Individuals who are involved with the criminal-legal system are among a particularly vulnerable population, represented by an increased TBI prevalence [5,6]. This population consists of individuals who interact with the criminal-legal system via police interactions, appearances before a judge in a criminal-legal setting, serving time in a correctional facility (e.g., jails, prisons, penitentiaries), parole, or probation [7]. A systematic review conducted by Durand and colleagues established a mean TBI prevalence of 46% among those involved with the criminal-legal system within their study [8]. Other studies have established TBI prevalences as high as 95% in the adult female prisoner population and 63.7% amongst their male counterparts [9,10]. Decreased abilities to regulate emotions and control impulses following TBIs were also found to increase aggressive behaviour among this population [11,12]. In turn, these changes may result in an increase in reoffending behaviour relative to individuals without TBI [13].

Rehabilitation is essential for the recovery of chronic conditions, such as TBI, to be able to regain lost skills and reduce the potential for symptom progression [14]. Barriers, such as being in a secure custodial setting, limits one’s ability to gain access to the full scope of services available to the public [15,16]. Therefore, it is imperative that the well-being of these specific individuals with TBI is considered when establishing rehabilitation due to their complex needs [15]. This issue can be mitigated through the implementation of compassionate care. As stated by Goetz and colleagues, compassion and compassionate care consists of the following: (1) awareness of a specific need or the existence of patient suffering, (2) physical and emotional experience of feeling “moved” through autonomic nervous system response, (3) evaluation of the care provider’s feelings, i.e., feeling “moved”, and their position in the context of being able to alleviate the suffering of the patient, (4) judgement and consideration for the patient’s specific needs to minimize suffering, and (5) the motivation to alleviate the identified suffering and the engagement in caregiving behaviour to do so [17]. Compassion is an essential part of treatment delivery as it holds potential to encourage hope, accountability, and patient resilience. It can also increase treatment adherence, patient satisfaction, clinician well-being, and overall positive health outcomes [18,19]. Compassion is distinguishable from trauma-informed and person-centred care as the latter two can exist on their own but are necessary components to compassionate care. Trauma-informed care, an aspect of care that considers a patient’s traumatic experience(s) to inform specific provision of care, can be identified as the first component in the definition provided by Goetz and colleagues [17,20]. Person-centred care establishes a care system that is responsive to the patient’s unique set of beliefs, goals, and concerns regarding their own rehabilitation [17,20]. This form of care is represented as an aspect of compassionate care through the judgement and consideration for patient-specific needs [17,20]. This is particularly vital for those who are involved with the criminal-legal system given their increased exposure and vulnerability to adverse experiences [10,21].

While rehabilitation and TBI continue to be explored in research, substantial gaps remain in literature surrounding the consideration and/or provision of compassionate care, especially in the context of individuals who intersect the criminal-legal system with TBI. This scoping review addressed these gaps by exploring the extent to which compassionate care is considered or integrated in rehabilitation for this specific population with TBI. Findings from this review provide a foundation to integrate compassionate care in rehabilitation for these individuals by understanding how compassionate care is currently provided in rehabilitation programs and/or services, including the health professionals who are involved and the location of services.

## Materials and methods

The methodology of the scoping review followed that of the published peer-review protocol established by Chan and colleagues, which is summarized below [22]. No formal protocol registration was taken for this review.

### Step one: Identifying the research question

The scoping review answered the research question “To what extent is compassionate care considered or provided in rehabilitation for individuals who are involved with the criminal-legal system with TBI?”

### Step two: Identifying the relevant studies

This scoping review updated the search strategy outlined by Chan and colleagues [6]. The search terms included: (1) traumatic brain injury, TBI, or cognitive impairment, (2) CJS, and (3) rehabilitation. The original search was co-created with an Information Specialist at the University Health Network (JB) and updated by a second Information Specialist at the University Health Network (CC) on January 10, 2025. The following databases were searched: MEDLINE, Embase, APA PsycINFO, Cochrane Central Register, Nursing and Allied Health Premium, Criminal Justice Abstracts, CINAHL Complete, and Applied Social Sciences Index & Abstracts. Grey literature was excluded as a separate search strategy is required to comprehensively and rigorously identify relevant studies. Table 1 highlights the terms TBI, CJS, and rehabilitation which helped guide the search strategy. The detailed search strategy can be referenced in S1 File.

**Table 1:** Definitions for traumatic brain injury, criminal justice system, and rehabilitation.

Concept	Definition
Traumatic Brain Injury (TBI)	“An alteration in brain function, or other evidence of brain pathology, caused by an external force.” [2]
Criminal Justice System (CJS)	Correctional Service Canada states individuals who are involved with the criminal-legal system are involved via the following ways: Policing/ police interaction, or Courts: Involvement in trials, prosecution, sentencing, or Corrections: involvement in the detention stage of the CJS, or Parole and probation [7].
Rehabilitation	A set of interventions designed to optimize functioning and reduce disability in individuals with health conditions in interaction with their environment,” or healthcare provider disciplines mentioned in the clinical practice guidelines established by Ontario Neurotrauma Foundation including the following: Case coordinator/ manager, Occupational Therapist, Physiotherapist, Physician/ Physiatrist, Psychologist/ Neuropsychologist, Psychiatrist/ Neuropsychiatrist, Social Worker, Speech-language Pathologist, Rehabilitation Support Personnel, and/or Therapeutic Recreationist [14,23].

### Step three: Study selection

Covidence was used for study selection and de-duplication. All articles were screened based on the following predetermined eligibility criteria, outlined by Chan and colleagues:

1. “Describe or document (a) rehabilitation programs or interventions or (b) services provided by healthcare providers or professional disciplines, as defined in Table 1, and
2. Include individuals (of any proportion) with TBI, and
3. Include individuals (of any proportion) who intersected with any part of the CJS, as defined in Table 1, and
4. Report primary research findings.” [6]

The following were excluded:

1. “Books and conference proceedings, or
2. Articles, gray literature, and reviews that are narrative, commentaries, or describe a theory or framework without reporting primary research findings, or
3. Articles that describe a sample including individuals with brain injury or individuals experiencing cognitive impairment without specific mention of TBI.” [6]

As per Chan et al., articles that “(a) included individual with brain injury or individuals experiencing cognitive impairment without specific mention of TBI or (b) were scoping or systematic reviews that met the above criteria were also considered for full-text review.” [6]

The title-abstract screening was initiated by a pilot screening of 20 randomly selected articles which were independently reviewed by two reviewers (MR, GC). An agreement rate of 80% or higher was established before continuing the abstract screening process. The agreement at the title and abstract screening stage was 78% for English language abstracts and 100% for non-English language abstracts.

At the full-text screen, two independent reviewers (MR, GC) reviewed the full-text articles based on the predetermined eligibility criteria. A pilot screen of 10% of randomly selected full-text articles was conducted until a minimum of 80% agreement was reached between the two reviewers. The resulting agreement at the full-text screen was 91% for English language articles. At both stages of the screening process, conflicts were resolved between the two reviewers by consensus or consultation with a third reviewer (VC).

### Step four: Charting the data

Microsoft Excel was used to chart the data of all included articles, including articles that met eligibility criteria from the scoping review by Chan and colleagues [6]. For this scoping review, the consideration and/or provision of compassionate care using the definition described by Goetz and colleagues was additionally charted [17]. A pilot data extraction of four randomly selected articles was conducted by two independent reviewers (MR, CM), ensuring a minimum agreement rate of 80% was met before moving to the remaining articles. There was an agreement rate of 96% between the reviewers (MR, CM).

### Step five: Collating, summarizing, and reporting the results

The reporting of the results was done in consideration of the research question: “To what extent is compassionate care considered or provided in rehabilitation for individuals who are involved with the criminal-legal system with TBI?”. A descriptive numerical summary of the data presented in the charting table (S2 File) and a qualitative thematic analysis was conducted to explore: (1) *who* provided compassionate care, (2) *where* the care was provided, and (3) *how* compassionate care was provided, based on the components of the compassionate care established by Goetz and colleagues [17].

## Results

**Fig 1:**
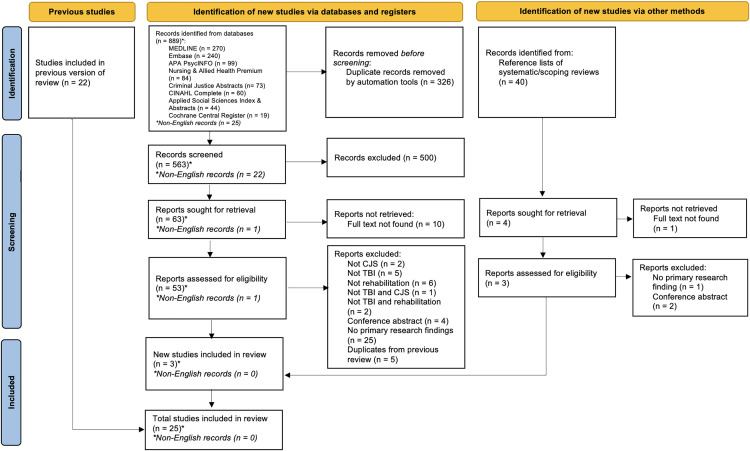
PRISMA flow diagram of updated CJS search.

From the updated search conducted on January 10, 2025, a total of 864 English language and 25 non-English language articles were identified. After de-duplication, 563 articles (English: n = 541; non-English: n = 22) were screened. Three primary research articles from the new search met eligibility criteria. Including the 22 articles from the published review by Chan et al., a total of 25 articles were considered for this current review [6]. Fig 1 presents the PRISMA Flow Chart documenting the study selection process.

Of the 25 articles, 48% (n = 12) were from the United States, 20% (n = 5) from the United Kingdom, 12% (n = 3) from Canada, 8% (n = 2) from Australia, 4% (n = 1) from New Zealand, 4% (n = 1) from Gaza, Egypt, Mexico, Honduras, and South Africa, and 4% (n = 1) from Poland. The articles in this review were published between 1991 and 2024, with 16% (n = 4) published before 2000, 24% (n = 6) published between 2000 and 2009, 32% (n = 8) published between 2010 and 2019, and 28% (n = 7) published in 2020 or later.

Six articles (24%) documented the consideration and/or provision of compassionate care. Linkworkers were identified as the primary healthcare providers in 50% (n = 3) of articles identified to incorporate compassionate care [24–26]. They were defined as psychology graduate students who gained experience through linkage programs before they began professional training into their specialized discipline (e.g., clinical psychology) [24]. Case managers were documented in two articles (33.3%) and were defined as professionals in resource facilitation for individuals on parole or probation [27,28]. The remaining 16.7% (n = 1) did not identify a rehabilitation team but defined them as trained through the Neurobehavioural Resource Project (NRP) [29]. Compassionate care was provided in secure settings (e.g., jails, prisons, etc) in 50% (n = 3) of articles through linkage programs [24–26]. It was also found to be provided in community-based settings in 100% (n = 6) of articles [24–29]. Table 2 presents the study characteristics.

**Table 2:** Study characteristics.

Characteristics	N (%)
**Country of study**	
United States [27–38]	12 (48%)
United Kingdom [24–26, 39, 40]	5 (20%)
Canada [41–43]	3 (12%)
Australia [44, 45]	2 (8%)
New Zealand [46]	1 (4%)
Gaza, Egypt, Mexico, Honduras, South Africa [47]	1 (4%)
Poland [48]	1 (4%)
**Study design**	
Cohort [24, 25, 27, 29–32, 34, 36–38, 44, 45, 47]	14 (56%)
Case studies [26, 39, 40, 41, 48]	5 (20%)
Randomized controlled trials [42, 43, 46]	3 (12%)
Non-randomized controlled trials [28]	1 (4%)
Cross-sectional [35]	1 (4%)
Before-after no control [33]	1 (4%)
**Sex and gender**	
Male and female [27,29–36,38,42,43,47]	13 (52%)
Males or men only [24–26,28,39–41,44–46,48]	11 (44%)
Unspecified [37]	1 (4%)
**Rehabilitation locations**	
Secure settings (e.g., Jails or Prison) [24–26,37, 46]	5 (20%)
Inpatient and outpatient rehabilitation [30,34,36,38–40,42,44,45,48]	10 (40%)
Residential rehabilitation [32,41]	2 (8%)
Community-based rehabilitation programs [26–29,33,35,42,43,47]	8 (32%)
Inpatient, outpatient, and secure settings [31]	1 (4%)
**Funding source of rehabilitation program/interventions**	
Federally funded [28, 36–38, 44]	5 (20%)
Implemented by a non-profit agency [25]	1 (4%)
Not reported [24,26,27,29–35,39–43,45–48]	19 (76%)
**CJS intersection**	
Corrections (e.g., “arrested,” “incarcerated,” “in custody,” “convicted,” “in prison,” “in jail,” “forensic history,” “criminal charges”) [25–27, 29, 31–34, 37, 39–41, 44–46]	15 (60%)
Parole/probation [26–28, 32, 37, 43, 44]	7 (28%)
Courts [26, 32, 35, 39, 40]	5 (20%)
Police [35, 41]	2 (8%)
More than one part of the CJS [26, 27, 32, 35, 37, 39–41, 43, 44]	10 (40%)
**Compassionate Care Characteristics (N = 6)**	***N (%)***
**Who provided compassionate care?**	
Linkworker [24–26]	3 (50%)
Case manager [27, 28]	2 (33.3%)
Not specified [29]	1 (16.7%)
**Where was compassionate care provided?**	
Secure settings (e.g., Jails or Prison) [24–26]	3 (50%)
Community-based rehabilitation programs [24–29]	6 (100%)

### How was compassionate care provided?

The provision of compassionate care was identified using the principles described by Goetz et al., which included (1) awareness of specific needs or existence of patient suffering, which was demonstrated through staff education, TBI screening tools, and mental health assessments; (2) judgements and consideration about the person who is suffering and the context of one’s position to aid in alleviating that suffering, which was demonstrated through patient consultation, patient-centred care frameworks, and case conferences involving multidisciplinary teams; and (3) motivation and engagement in caregiving to help minimize or alleviate suffering, which was demonstrated through collaboration of multidisciplinary professionals, maintaining one-on-one contact with patients throughout intervention, and patient-specific program modifications [17,24–29].

### Awareness of patient suffering

Awareness of patient suffering was demonstrated through the awareness of TBI, TBI-related impairments, comorbid conditions, and others, through TBI screening (e.g., using the HELPS Brain Injury Screening Tool), assessment tools (e.g., using the Patient Health Questionnaire-9), as well as specialized staff education [24,27]. Linkage programs and case management programs trained staff to work with individuals who intersect the criminal-legal system with TBI [24–28]. Healthcare professionals were not explicitly identified by Ylvisaker et al., but were documented to be trained to work with individuals with TBI through the NRP [29]. For example, staff were documented to have gone through apprenticeship programs to develop awareness on TBI-related barriers to the NRP [29]. Case management described in Ahlers and colleagues also included assessments for trauma history, substance use disorder, and behavioural health diagnoses [27]. Offense and medical records were also retrieved as part of the linkage programs [24–26]. Awareness was further demonstrated in Trexler and Parrott’s Modified Resource Facilitation (RF) program through evaluation of cognitive and neurobehavioural functions, substance abuse, and level of disability [28]. In the traditional RF program, family and social support, pain, mobility, personality, emotional functioning, and vocational preferences and barriers were additionally evaluated [28]. Specific screening tools were not mentioned in Ylvisaker and colleagues but were focused on awareness of substance abuse in addition to TBI [29].

### Judgement and consideration of patient suffering

Judgement and consideration of patient suffering were demonstrated through person-centred and individualized rehabilitation plans. Person-centred care, as one of the “Ten Principles Governing Delivery of Services and Supports Within the Neurobehavioural Resource Project”, was discussed by Ylvisaker et al [29]. All rehabilitation programs documented patient consultation to determine what aspects of recovery would be addressed first [24–29]. Linkage programs incorporated one-on-one meetings setting personal rehabilitative goals and established plans to reach these goals [24–26]. Case management described by Trexler & Parrott included “local support network community assessments”, described as consulting with the patient to identify specific rehabilitative needs in which case managers evaluated and identified available community resources that targeted those needs [28]. Consideration of results from screening tools and mental health assessments were documented in linkage programs and case management to inform these judgements [24–28]. The NRP demonstrated a person-centred approach and allowed patients to pave their own path for rehabilitation [29]. Through this process, NRP staff considered the situation of each patient and ensured realistic rehabilitative goals were set and that the services provided would contribute to the patient’s rehabilitative success [29]. For example, one principle of the NRP was to ensure proposed interventions and supports were organized around “personally meaningful activities” [29]. Judgement and consideration were demonstrated as all rehabilitation programs involved the monitoring of patients throughout the interventions and included the evaluations of program effectiveness [24–29]. For example, patients in the linkage programs and case management described in Trexler & Parrott still received the programs’ rehabilitative effects, even after custodial release, by keeping in contact with care providers who made changes to rehabilitation programs when needed [24–26,28]. Case conferences with RF teams to assess the patient’s level and area of need was also identified in RF interventions [28]. These conferences were held monthly with subsequent documentation to assess progress throughout the program [28].

### Motivation to alleviate patient suffering

Motivation and actions to alleviate suffering was demonstrated through the multidisciplinary nature of the rehabilitation interventions and collaboration of health professionals from additional agencies and services. For example, linkage programs revolved around assisting patients form relationships with agencies within their custodial settings in preparation for community reintegration [24–26]. For specific patients, linkworkers shared the patient’s needs and accommodations to vocational agencies to find employment meeting these needs. Case management involved connecting patients with “vocational placement services” and job coaching for a 90-day period and directed them to the Indiana Vocational Rehabilitation Services (IVRS) for additional assistance [28]. Additionally, linkage programs were documented to help those who lost their housing while serving a custodial sentence by connected them to housing agencies. Brain injury awareness training for the patients, family, correctional staff, etc., were also part of linkage program and case management [24–28]. Patient education was tailored to the patient and focused on the development of adaptive coping strategies [26]. NRP focused on establishing positive daily routines for patients struggling with cognitive flexibility [29]. Case management consisted of weekly TBI education and life skills groups for patients and provided them with “TBI notebooks” containing information regarding medical providers, employers, etc [28]. On top of establishing connections with services and service providers in secure and community-based settings, care providers also maintained contact with patients throughout the rehabilitation process [24–29]. In linkage programs, multi-agency review meetings in custodial or community settings were held and progress reports were shared with agencies involved in the patient’s rehabilitation (e.g., general physician, employer, etc) [24–26]. In Trexler and Parrott, case management involved biweekly meetings with patients for a 12-month period [28]. In the modified RF program, case managers met with patients on an as-needed basis [28]. The patients’ progress were tracked every six months in the case management intervention described by Ahlers et al [27]. As patients were monitored, modifications to the rehabilitative program were made based on patient progress [27,28]. The NRP intervention demonstrated this through the alleviation of patient supports as they made progress through the program to help achieve patient self-efficacy [29]. Additionally, Trexler & Parrott’s case management provided TBI wallet cards containing patient-specific TBI information [28]. The service delivery of case management in Ahlers and colleagues was adjusted for patients and provided services both in-person and over-the-phone [27].

In summary, awareness and judgement of patient suffering follow similar methods among the programs despite setting differences (i.e., secure, community-based, etc). An important distinction of how motivation to alleviate suffering is represented across the different settings should be noted. Among secure settings, linkage programs focused on connection building with community services in preparation for life upon release [24–26]. Case management and the NRP intervention showcased this motivation through the implementation of rehabilitation plans and the monitoring of patient success throughout these programs [27–29].

## Discussion

There is currently limited consideration for compassionate care in rehabilitation for individuals who are involved with the criminal-legal system with TBI. Six articles (24%) in this review considered/integrated compassionate care [24–29]. Awareness of suffering was demonstrated through staff education, TBI screening tools, and mental health assessments; judgement and consideration were demonstrated through case conferences, formulation of rehabilitative plans, monitoring of the plan’s efficacy for recovery; and motivation to alleviate suffering was documented through brain injury awareness training for patients and family, advocating for the patient, and acting as a link between patients and agencies within the community [24–29]. Findings from this review highlight the following opportunities to integrate compassionate care in rehabilitation for individuals who intersect the criminal-legal system with TBI: (1) routine TBI and CJS-related education and TBI screening to increase awareness of patient suffering, (2) person-centred frameworks to address judgement and consideration of patient suffering, and (3) establish relationships between patients and service providers and monitor progress throughout rehabilitation to alleviate patient suffering. Recommendation for further research on compassionate care and rehabilitation for these individuals were also identified.

Awareness of patient suffering was demonstrated through staff education on brain injury awareness, and in 83.3% (n = 5) of articles, staff education was specific to those involved with the criminal-legal system with TBI [24–28]. Implementing staff education to motivate and induce confidence in healthcare providers to provide care that effectively targets patients’ unique needs has been previously highlighted in the literature, and may be critical in supporting underserved populations [49]. TBI impacts individuals’ abilities to critically think, control impulses, emotionally regulate, etc [12]. In this specific population, this may look like increased aggression and violence, and decreased reasoning leading to recidivism in custody or within the community [11,13]. Therefore, it is crucial for care providers to be educated on the impacts of TBI, considering it may look like increased disobedience towards correctional staff, increased irritability, emotion dysregulation, and reoffending behaviour [11–13]. Staff education aids care providers in building awareness of symptoms and barriers associated with TBI, helping them identify signs and/or features of TBI in patients, as well as confidently and comfortably provide rehabilitative services [49]. Thus, education for participating care providers such as family, correctional staff, etc. informs them of patient suffering and promotes understanding of specific behaviour that would otherwise be perceived as threatening or punishable [12,24–28]. Similarly, the use of TBI and related screening tools also increased awareness of patient suffering. In most articles, screening tools, including those beyond TBI, such as mental health assessments, promoted the awareness of TBI symptoms, the extent to which they are disabling to the patient, and other comorbid conditions that further impede one’s well-being and health [24–28,50]. Collectively, findings suggest that incorporating routine TBI and CJS-related education and TBI screening in rehabilitative programs holds the potential to increase awareness of patient suffering to enable the integration of compassionate care into rehabilitation for individuals who intersect the criminal-legal system with TBI.

Person-centred approaches to rehabilitations may support judgements and considerations made by care providers of patient-specific needs to minimize suffering. This may involve the integration of screening assessment results, medical and offense history, and other evaluations to inform the patient’s state and extent of rehabilitation that is needed. In the NRP discussed in Ylvisaker and colleagues, person-centred care was shown by allowing the patient to guide their projects [29]. This enabled patients to take control of their rehabilitation journey, which has shown to increase compliance in more difficult patients [18]. Additionally, the use of interviews to retrieve first-hand experiences of the effects of TBI can also help establish more person-centred rehabilitative approaches. Person-centred rehabilitation was further demonstrated through the establishment of strong interpersonal bonds between the patient and healthcare provider(s), and consideration for the patient’s needs. For example, the establishment of case conferences in which multidisciplinary teams discuss the patient’s needs and possibilities of rehabilitation was identified [28]. Additionally, judgement and consideration were highlighted by the recognition of the intersectionality of underserved populations. Consideration for patients that required housing accommodations after long-term custodial sentences or those who were homeless upon entering the justice system, etc. were highlighted in case management and linkage services [24–28]. Judgments on the patient’s situation and consideration of what they have to say is imperative for person-centred, compassionate care.

Resource facilitation may present opportunities to further enhance compassionate care by motivating care providers to alleviate the suffering of patients. All rehabilitation services identified to provide compassionate care in this review helped patients gain access to resources from secure settings or within the community [24–29]. These results resemble the literature on the inequitable service provision, in which underserved populations, such as those who intersect the criminal-legal system, do not have equal access to care as the public [51]. Linkage services supported individuals in building connections with services outside the correctional facility to re-integrate back into the community. This indicates the importance of resource facilitation as means for providing compassionate care, as it explicitly addresses the disparities involved with being incarcerated and works around them to provide inclusive and considerate care. Similarly, case management pointed out resources within the community for patients on parole or probation [27,28]. Finally, continuous one-on-one relationships between patients and healthcare providers throughout the interventions was also critical, and has been demonstrated in a separate review to form strong provider-patient relationships to further address motivation and action to alleviate patient suffering as part of compassionate care [24–29,49].

Finally, this scoping review highlights an extensive research gap regarding compassionate care in rehabilitation for individuals who are involved with the criminal-legal system with TBI, specifically in *how* it may be routinely integrated and its impact (positive or negative) on individuals. For example, while this review identified that TBI screening and the inclusion of other health assessments may enhance care providers’ awareness of the patients’ suffering, it is critical to also consider potential negative impacts of being identified as an individual with a TBI (e.g., public or self-stigma) or how this information may be used against them in settings outside of healthcare (e.g., criminal-legal) [52,53]. For example, research suggests those with TBI and other cognitive disabilities are at higher risk of confabulation and are less likely to be perceived as credible by jurors [54,55]. Similarly, while the identification of other health conditions, such as mental health challenges, may inform patient-centred care, it is critical to also consider the impact of this diagnosis on patients’ access to other health services, as some may have exclusionary criteria based on mental health or substance use. Furthermore, it is crucial to consider *why* compassionate care is underrepresented when treating this specific population with TBI. While lack of TBI-specific education may play a role in the under-integration of compassionate care, other limiting factors such stigma from healthcare providers and/or policymakers and limited institutional support should be explored further. Underserved populations may also be underrepresented in literature due to increased vulnerabilities [56]. Finally, collaboration with both service providers and service users is critical to inform the co-creation of education content to support compassionate care [49,57]. Consideration of service users in the development of TBI education opens to door for more informed and person-centred rehabilitation frameworks that are tailored for specific populations with TBI (e.g., individuals who are involved with the criminal-legal system). The implementation of TBI education in all settings individuals with TBI may intersect, such as the criminal-legal system, shows potential for improving knowledge on the impact of TBI on the individual [58]. Furthermore, TBI education that is specific to these individuals may increase awareness of TBI-associated trauma promoting (1) awareness of suffering, (2) judgement and consideration of suffering, and (3) motivation and trauma-informed action to alleviate suffering.

### Strengths and limitations

A limitation of this review is the use of English search terms (e.g., traumatic brain injury, rehabilitation, etc.), which may limit the identification of potentially relevant non-English language articles on compassionate care. Similarly, many of the included articles/studies in this review were published in Western countries, with most of them originating from the United States. As such, findings from this review may represent a Western perspective on compassionate care and may not be generalizable to other countries. The definition of compassionate care established by Goetz and colleagues also parallels this limitation, as it was established under a Western context [17]. Thus, the use of this definition does not consider potential differences in representation of compassionate care across different languages and cultures. Furthermore, this review only included published peer-reviewed articles, excluding grey literature and other non-peer-reviewed papers listed in the exclusion criteria. It is probable that other rehabilitation services provided in the community setting exist but do not participate in research activities and thus, may be missed in this review. We acknowledge this limitation and that there may be a portion of custodial and community-based rehabilitation programs that have not been published for peer-review.

Nonetheless, there are notable strengths to this review. This scoping review is the first, to the best of our knowledge, to explore compassionate care in rehabilitation for individuals who are involved with the criminal-legal system with TBI. It also ensured methodological rigour, which has been highlighted as a limitation in the rehabilitation scoping review by Colquhoun et al. This was done by updating the peer-reviewed scoping review by Chan and colleagues, which has also followed a peer-reviewed protocol [6,59]. Finally, this review explored compassionate care through the components established by Goetz and colleagues to understand the presence or absence of specific attributes of compassion to provide a foundation to integrate compassionate care in rehabilitation for these individuals [17].

## Conclusion

This scoping review explored compassionate care in rehabilitation for individuals who intersect the criminal-legal system with TBI. Findings showed a lack of routine consideration of compassionate care in rehabilitation for this population, as a limited number of articles considered or provided compassionate care. However, it also highlighted that specific rehabilitation programs, such as linkage program, case management, and NRP interventions provide opportunities to integrate compassionate care through (1) routine TBI and CJS-related education and TBI screening to increase awareness of patient suffering, (2) person-centred frameworks to address judgement and consideration of patient’s suffering, and (3) establish relationships between patients and service providers and monitor progress throughout rehabilitation to alleviate patient suffering. Further research on how compassionate care may be routinely integrated and its impact (positive or negative) on individuals should be explored. The association between compassionate care components and rehabilitation outcomes should also be investigated to inform opportunities for institutional changes, as well as the co-creation of education content with both service users and service providers to address population-specific goals (e.g., successful community reintegration, reduced recidivism, etc) and support compassionate care.

## Supporting information

S1 File Search strategy and supporting documentation. (DOCX)

S2 File Charting table of compassionate care and of newly identified articles from updated search. *BISI*: Brain Injury Screening Index; *CHAT*: Comprehensive Health Assessment Tool; *DUI*: Driving Under the Influence; *GAD-7*: Generalized Anxiety Disorder-7; *M2PI*: Mayo-Portland Adaptability Inventory-4 Participation Index; *MPAI-4*: Mayo-Portland Adaptability Inventory-4; *NRP*: Neurobehavioural Resource Project; *PHQ-9*: Patient Health Questionnaire-9; *RF*: Resource Facilitation.
(DOCX)
