# Supplementary material for: Exploring compassionate care in rehabilitation among individuals who are involved with the criminal-legal system with traumatic brain injury: A scoping review
Source: PLoS One. 2026 Jun 24;21(6):e0341381. doi: 10.1371/journal.pone.0341381 (PMC13293406; doi:10.1371/journal.pone.0341381)
Supplement: S1 File — (DOCX) [file pone.0341381.s001.docx]

**Search Strategy and Supporting Documentation (TBI & CJS)**

**Submitted: 2025-01-10**

**UPDATE PRE-DUPLICATE REMOVAL RESULTS (for PRISMA reporting)**

Last Search Date: July 6, 2021

Current Update Date: January 10, 2025

*TOTAL Results: 754 citations*

- MEDLINE(R) ALL: 135 citations
- Embase Classic+Embase: 240 citations
- Cochrane Central Register of Controlled Trials: 19 citations
- APA PsycInfo: 99 citations
- CINAHL Ultimate: 60 citations
- Criminal Justice Abstracts: 73 citations
- Nursing and Allied Health Premium: 84 citations
- Applied Social Sciences Index & Abstracts: 44 citations

*English Results: 731 citations*

- MEDLINE(R) ALL: 133 citations
- Embase Classic+Embase: 235 citations
- Cochrane Central Register of Controlled Trials: 19 citations
- APA PsycInfo: 87 citations
- CINAHL Ultimate: 60 citations
- Criminal Justice Abstracts: 70 citations
- Nursing and Allied Health Premium: 83 citations
- Applied Social Sciences Index & Abstracts: 44 citations

*Other Language Results: 23 citations*

- MEDLINE(R) ALL: 2 citations
- Embase Classic+Embase: 5 citations
- Cochrane Central Register of Controlled Trials: 0 citations
- APA PsycInfo: 12 citations
- CINAHL Ultimate: 0 citations
- Criminal Justice Abstracts: 3 citations
- Nursing and Allied Health Premium: 1 citation
- Applied Social Sciences Index & Abstracts: 0 citations

**SEARCH STRATEGIES – Update Jan 10, 2025 (Last search date July 6, 2021)**

**Database:** Ovid MEDLINE(R) ALL <1946 to January 06, 2025>

| **#** | **Query** | **Results from 10 Jan 2025** |
| --- | --- | --- |
| 1 | exp PRISONS/ | 12,380 |
| 2 | exp PRISONERS/ | 19,376 |
| 3 | exp CRIMINALS/ | 6,708 |
| 4 | Criminal Law/ or Jurisprudence/ | 35,544 |
| 5 | Judicial Role/ | 3,518 |
| 6 | (jurisprudenc* or ligitat*).tw,kf. | 7,072 |
| 7 | (legal adj (system? or servic*)).tw,kf. | 3,061 |
| 8 | (prisoner* or prison? or imprison*).tw,kf. | 23,285 |
| 9 | (inmate* or convict* or criminal* or offender?).tw,kf. | 50,011 |
| 10 | (correctional adj2 (setting? or service? or units or unit or facility or facilities or institution* or centre* or center*)).tw,kf. | 2,975 |
| 11 | (penal adj2 (setting? or service? or units or unit or facility or facilities or institution* or centre* or center*)).tw,kf. | 181 |
| 12 | (jail* or penitentiar* or gaol*).tw,kf. | 5,797 |
| 13 | incarcerat*.tw,kf. | 16,451 |
| 14 | (detain* or detention?).tw,kf. | 6,487 |
| 15 | parole?.tw,kf. | 964 |
| 16 | probation*.tw,kf. | 2,055 |
| 17 | felon*.tw,kf. | 901 |
| 18 | Police/ | 7,258 |
| 19 | (police or policing).tw,kf. | 20,598 |
| 20 | law enforce*.tw,kf. | 7,063 |
| 21 | forensic*.tw,kf. | 60,620 |
| 22 | forensic psychiatry/ or "commitment of mentally ill"/ or insanity defense/ | 16,169 |
| 23 | (correctional or forensic).jw. | 40,127 |
| 24 | or/1-23 | 224,118 |
| 25 | "Physical and Rehabilitation Medicine"/ | 3,712 |
| 26 | exp rehabilitation/ | 373,159 |
| 27 | rehab*.tw,kf,jw. | 310,060 |
| 28 | telerehab*.tw,kf,jw. | 2,814 |
| 29 | neurorehab*.tw,kf,jw. | 10,411 |
| 30 | rh.fs. | 214,637 |
| 31 | (physiatrist? or physiatry).tw,kf. | 1,969 |
| 32 | Rehabilitation Centers/ | 8,826 |
| 33 | occupational therapy/ | 15,698 |
| 34 | (occupational adj therap*).tw,kf,jw. | 20,253 |
| 35 | physical therapy specialty/ | 3,118 |
| 36 | (physical adj therap*).tw,kf,jw. | 36,884 |
| 37 | physiotherap*.tw,kf,jw. | 46,574 |
| 38 | physio-therapist*.tw,kf,jw. | 15 |
| 39 | Speech-Language Pathology/ | 4,008 |
| 40 | (speech adj2 (therap* or patholog*)).tw,kf,jw. | 14,025 |
| 41 | Neuropsychology/ | 2,709 |
| 42 | Neuropsycholog*.tw,kf,jw. | 93,493 |
| 43 | Nutritionists/ | 1,974 |
| 44 | (Nutritionist? or Dietician?).tw,kf,jw. | 6,702 |
| 45 | (therap* adj recreation*).tw,kf,jw. | 236 |
| 46 | child life specialist?.tw,kf. | 229 |
| 47 | play therapy/ | 1,218 |
| 48 | (play adj therap*).tw,kf. | 581 |
| 49 | Respite Care/ | 1,108 |
| 50 | respite.tw,kf. | 2,370 |
| 51 | Case Managers/ | 330 |
| 52 | Case Management/ | 10,842 |
| 53 | case manag*.tw,kf. | 16,574 |
| 54 | exp Social Work/ | 19,106 |
| 55 | social work*.tw,kf,jw. | 26,117 |
| 56 | Forensic Nursing/ | 582 |
| 57 | (nurse? or nursing).tw,kf,jw. | 877,123 |
| 58 | Community Integration/ | 470 |
| 59 | (integrat* or reintegrat* or re-integrat* or reentry or re-entry or resettle* or re-settle*).tw,kf. | 855,705 |
| 60 | Aftercare/ | 13,918 |
| 61 | (Aftercare or "after care").tw,kf. | 5,855 |
| 62 | Transitional Care/ | 1,418 |
| 63 | "transitional care".tw,kf. | 2,648 |
| 64 | or/25-63 | 2,508,899 |
| 65 | exp Brain Injuries/ | 87,579 |
| 66 | exp Brain Injuries, Traumatic/ | 28,134 |
| 67 | exp Brain Concussion/ | 13,516 |
| 68 | Craniocerebral Trauma/ | 24,120 |
| 69 | tbi*2.tw,kf. | 39,302 |
| 70 | mtbi*2.tw,kf. | 4,650 |
| 71 | concuss*.tw,kf. | 14,490 |
| 72 | postconcuss*.tw,kf. | 1,845 |
| 73 | ((head* or brain* or cerebr* or crani* or skull* or intracran*) adj2 (injur* or trauma* or damag* or wound* or swell* or oedema* or edema* or fracture* or contusion* or pressur*)).tw,kf,jw. | 222,367 |
| 74 | ((brain* or cerebr* or intracerebr* or crani* or intracran* or head* or subdural* or epidural* or extradural*) adj (haematoma* or hematoma* or hemorrhag* or haemorrhag* or bleed*)).tw,kf. | 70,174 |
| 75 | exp cognition disorders/ | 126,227 |
| 76 | ((cogniti* or neurocogniti*) adj2 (impair* or dysfunction* or disorder* or declin*)).tw,kf. | 178,605 |
| 77 | or/65-76 | 537,098 |
| 78 | 24 and 64 | 23,729 |
| 79 | 24 and 77 | 4,980 |
| 80 | 24 and 64 and 77 | 757 |
| 81 | 80 not (exp animals/ not humans.sh.) | 756 |
| 82 | limit 81 to english language | 706 |
| 83 | 81 not 82 | 50 |
| 84 | ("20210706" or "20210707" or "20210708" or "20210709" or 2021071* or 2021072* or 2021073* or 202108* or 202109* or 20211* or 2022* or 2023* or 2024* or 2025*).dt,ez,da. | 6,172,099 |
| 85 | 82 and 84 | 133 |
| 86 | 83 and 84 | 2 |

*******************************

**Database:** Embase Classic+Embase <1947 to 2025 January 09>

| **#** | **Query** | **Results from 10 Jan 2025** |
| --- | --- | --- |
| 1 | exp PRISON/ | 4,610 |
| 2 | PRISONER/ | 21,214 |
| 3 | Offender/ | 19,794 |
| 4 | Criminal Justine/ or Jurisprudence/ | 30,862 |
| 5 | legal procedure/ or probation/ | 1,974 |
| 6 | (jurisprudenc* or ligitat*).tw,kw. | 2,635 |
| 7 | (legal adj (system? or servic*)).tw,kw. | 3,689 |
| 8 | (prisoner* or prison? or imprison*).tw,kw. | 28,809 |
| 9 | (inmate* or convict* or criminal* or offender?).tw,kw. | 64,223 |
| 10 | (correctional adj2 (setting? or service? or units or unit or facility or facilities or institution* or centre* or center*)).tw,kw. | 3,524 |
| 11 | (penal adj2 (setting? or service? or units or unit or facility or facilities or institution* or centre* or center*)).tw,kw. | 310 |
| 12 | (jail* or penitentiar* or gaol*).tw,kw. | 7,169 |
| 13 | incarcerat*.tw,kw. | 21,028 |
| 14 | (detain* or detention?).tw,kw. | 8,928 |
| 15 | parole?.tw,kw. | 1,087 |
| 16 | probation*.tw,kw. | 2,695 |
| 17 | felon*.tw,kw. | 1,060 |
| 18 | exp police/ or detention/ | 20,008 |
| 19 | (police or policing).tw,kw. | 26,619 |
| 20 | law enforce*.tw,kw. | 8,502 |
| 21 | forensic*.tw,kw. | 86,250 |
| 22 | exp forensic medicine/ | 65,583 |
| 23 | (correctional or forensic).jx. | 60,467 |
| 24 | or/1-23 | 302,890 |
| 25 | rehabilitation medicine/ or physical medicine/ | 20,347 |
| 26 | exp rehabilitation/ | 553,187 |
| 27 | rehab*.tw,kw,jx. | 463,511 |
| 28 | telerehab*.tw,kw,jx. | 2,996 |
| 29 | neurorehab*.tw,kw,jx. | 15,528 |
| 30 | rh.fs. | 183,312 |
| 31 | (physiatrist? or physiatry).tw,kw. | 3,445 |
| 32 | Rehabilitation Center/ | 22,238 |
| 33 | occupational therapy/ | 31,594 |
| 34 | (occupational adj therap*).tw,kw,jx. | 37,150 |
| 35 | exp physiotherapy/ | 129,155 |
| 36 | (physical adj therap*).tw,kw,jx. | 64,332 |
| 37 | physiotherap*.tw,kw,jx. | 84,645 |
| 38 | physio-therapist*.tw,kw,jx. | 39 |
| 39 | "speech and language rehabilitation"/ | 1,533 |
| 40 | (speech adj2 (therap* or patholog*)).tw,kw,jx. | 20,779 |
| 41 | Neuropsychology/ | 21,772 |
| 42 | Neuropsycholog*.tw,kw,jx. | 130,485 |
| 43 | Dietitian/ | 20,208 |
| 44 | (Nutritionist? or Dietician?).tw,kw,jx. | 13,343 |
| 45 | (therap* adj recreation*).tw,kw,jx. | 407 |
| 46 | child life specialist?.tw,kw. | 554 |
| 47 | play therapy/ | 2,182 |
| 48 | (play adj therap*).tw,kw. | 914 |
| 49 | Respite Care/ | 1,349 |
| 50 | respite.tw,kw. | 3,230 |
| 51 | Case Manager/ | 2,705 |
| 52 | Case Management/ | 14,281 |
| 53 | case manag*.tw,kw. | 22,069 |
| 54 | Social Work/ | 32,911 |
| 55 | social work*.tw,kw,jx. | 37,891 |
| 56 | Forensic Nursing/ | 608 |
| 57 | (nurse? or nursing).tw,kw,jx. | 989,819 |
| 58 | Community Integration/ or community reintegration/ | 2,377 |
| 59 | (integrat* or reintegrat* or re-integrat* or reentry or re-entry or resettle* or re-settle*).tw,kw. | 1,024,160 |
| 60 | Aftercare/ | 9,959 |
| 61 | (Aftercare or "after care").tw,kw. | 8,646 |
| 62 | Transitional Care/ | 6,497 |
| 63 | "transitional care".tw,kw. | 3,871 |
| 64 | or/25-63 | 3,139,414 |
| 65 | exp Brain Injury/ | 243,090 |
| 66 | exp traumatic brain injury/ | 75,996 |
| 67 | Brain concussion/ or postconcussion syndrome/ | 12,175 |
| 68 | head injury/ | 63,246 |
| 69 | tbi*2.tw,kw. | 63,398 |
| 70 | mtbi*2.tw,kw. | 7,089 |
| 71 | concuss*.tw,kw. | 20,435 |
| 72 | postconcuss*.tw,kw. | 2,500 |
| 73 | ((head* or brain* or cerebr* or crani* or skull* or intracran*) adj2 (injur* or trauma* or damag* or wound* or swell* or oedema* or edema* or fracture* or contusion* or pressur*)).tw,kw,jx. | 307,384 |
| 74 | ((brain* or cerebr* or intracerebr* or crani* or intracran* or head* or subdural* or epidural* or extradural*) adj (haematoma* or hematoma* or hemorrhag* or haemorrhag* or bleed*)).tw,kw. | 106,638 |
| 75 | exp cognitive defect/ | 683,215 |
| 76 | ((cogniti* or neurocogniti*) adj2 (impair* or dysfunction* or disorder* or declin*)).tw,kw. | 257,157 |
| 77 | or/65-76 | 1,231,655 |
| 78 | 24 and 64 | 32,218 |
| 79 | 24 and 77 | 10,159 |
| 80 | 24 and 64 and 77 | 1,562 |
| 81 | 80 not medline.cr. | 1,215 |
| 82 | 81 not (((rat or rats or mouse or mice or swine or porcine or murine or sheep or lambs or pigs or piglets or rabbit or rabbits or cat or cats or dog or dogs or cattle or bovine or monkey or monkeys or trout or marmoset$1).ti. and animal experiment/) or (Animal experiment/ not (human experiment/ or human/))) | 1,206 |
| 83 | limit 82 to english language | 1,121 |
| 84 | 82 not 83 | 85 |
| 85 | limit 83 to dc=20210706-20250110 | 235 |
| 86 | limit 84 to dc=20210706-20250110 | 5 |

***************************

**Database:** Cochrane Central Register of Controlled Trials <2014 to Present>

| **#** | **Query** | **Results from 10 Jan 2025** |
| --- | --- | --- |
| 1 | exp PRISONS/ | 200 |
| 2 | exp PRISONERS/ | 469 |
| 3 | exp CRIMINALS/ | 200 |
| 4 | Criminal Law/ or Jurisprudence/ | 179 |
| 5 | Judicial Role/ | 23 |
| 6 | (jurisprudenc* or ligitat*).tw,kw. | 95 |
| 7 | (legal adj (system? or servic*)).tw,kw. | 106 |
| 8 | (prisoner* or prison? or imprison*).tw,kw. | 1,160 |
| 9 | (inmate* or convict* or criminal* or offender?).tw,kw. | 2,485 |
| 10 | (correctional adj2 (setting? or service? or units or unit or facility or facilities or institution* or centre* or center*)).tw,kw. | 323 |
| 11 | (penal adj2 (setting? or service? or units or unit or facility or facilities or institution* or centre* or center*)).tw,kw. | 4 |
| 12 | (jail* or penitentiar* or gaol*).tw,kw. | 425 |
| 13 | incarcerat*.tw,kw. | 992 |
| 14 | (detain* or detention?).tw,kw. | 259 |
| 15 | parole?.tw,kw. | 135 |
| 16 | probation*.tw,kw. | 281 |
| 17 | felon*.tw,kw. | 51 |
| 18 | Police/ | 134 |
| 19 | (police or policing).tw,kw. | 861 |
| 20 | law enforce*.tw,kw. | 181 |
| 21 | forensic*.tw,kw. | 610 |
| 22 | forensic psychiatry/ or "commitment of mentally ill"/ or insanity defense/ | 156 |
| 23 | (correctional or forensic).jw. | 160 |
| 24 | or/1-23 | 5,867 |
| 25 | "Physical and Rehabilitation Medicine"/ | 37 |
| 26 | exp rehabilitation/ | 56,071 |
| 27 | rehab*.tw,kw,jw. | 64,155 |
| 28 | telerehab*.tw,kw,jw. | 1,554 |
| 29 | neurorehab*.tw,kw,jw. | 1,895 |
| 30 | rh.fs. | 23,684 |
| 31 | (physiatrist? or physiatry).tw,kw. | 250 |
| 32 | Rehabilitation Centers/ | 383 |
| 33 | occupational therapy/ | 1,039 |
| 34 | (occupational adj therap*).tw,kw,jw. | 4,533 |
| 35 | physical therapy specialty/ | 159 |
| 36 | (physical adj therap*).tw,kw,jw. | 12,397 |
| 37 | physiotherap*.tw,kw,jw. | 25,895 |
| 38 | physio-therapist*.tw,kw,jw. | 7 |
| 39 | Speech-Language Pathology/ | 129 |
| 40 | (speech adj2 (therap* or patholog*)).tw,kw,jw. | 2,162 |
| 41 | Neuropsychology/ | 39 |
| 42 | Neuropsycholog*.tw,kw,jw. | 11,772 |
| 43 | Nutritionists/ | 87 |
| 44 | (Nutritionist? or Dietician?).tw,kw,jw. | 2,338 |
| 45 | (therap* adj recreation*).tw,kw,jw. | 26 |
| 46 | child life specialist?.tw,kw. | 50 |
| 47 | play therapy/ | 98 |
| 48 | (play adj therap*).tw,kw. | 275 |
| 49 | Respite Care/ | 16 |
| 50 | respite.tw,kw. | 194 |
| 51 | Case Managers/ | 22 |
| 52 | Case Management/ | 922 |
| 53 | case manag*.tw,kw. | 3,254 |
| 54 | exp Social Work/ | 282 |
| 55 | social work*.tw,kw,jw. | 2,692 |
| 56 | Forensic Nursing/ | 3 |
| 57 | (nurse? or nursing).tw,kw,jw. | 65,527 |
| 58 | Community Integration/ | 21 |
| 59 | (integrat* or reintegrat* or re-integrat* or reentry or re-entry or resettle* or re-settle*).tw,kw. | 40,616 |
| 60 | Aftercare/ | 1,227 |
| 61 | (Aftercare or "after care").tw,kw. | 1,496 |
| 62 | Transitional Care/ | 151 |
| 63 | "transitional care".tw,kw. | 653 |
| 64 | or/25-63 | 243,958 |
| 65 | exp Brain Injuries/ | 3,819 |
| 66 | exp Brain Injuries/ | 3,819 |
| 67 | exp Brain Concussion/ | 716 |
| 68 | Craniocerebral Trauma/ | 451 |
| 69 | tbi*2.tw,kw. | 4,150 |
| 70 | mtbi*2.tw,kw. | 501 |
| 71 | concuss*.tw,kw. | 1,186 |
| 72 | postconcuss*.tw,kw. | 350 |
| 73 | ((head* or brain* or cerebr* or crani* or skull* or intracran*) adj2 (injur* or trauma* or damag* or wound* or swell* or oedema* or edema* or fracture* or contusion* or pressur*)).tw,kw,jw. | 16,504 |
| 74 | ((brain* or cerebr* or intracerebr* or crani* or intracran* or head* or subdural* or epidural* or extradural*) adj (haematoma* or hematoma* or hemorrhag* or haemorrhag* or bleed*)).tw,kw. | 10,230 |
| 75 | exp cognition disorders/ | 8,794 |
| 76 | ((cogniti* or neurocogniti*) adj2 (impair* or dysfunction* or disorder* or declin*)).tw,kw. | 24,659 |
| 77 | or/65-76 | 54,292 |
| 78 | 24 and 64 | 1,283 |
| 79 | 24 and 77 | 136 |
| 80 | 24 and 64 and 77 | 53 |
| 81 | "[https://clinicaltrials.gov*".so](https://urldefense.com/v3/__https://clinicaltrials.gov**A22.so__;KiU!!CjcC7IQ!KEyyMCdUwDVR4bHWSotIUaQMmv2K_8VcuH53rmagNr1NcYEEz-_Luy3S7RU-CByIo_dYzQZihrabsA_VuefevA$). | 8 |
| 82 | "[http://www.who.int/trialsearch*".so](https://urldefense.com/v3/__http://www.who.int/trialsearch**A22.so__;KiU!!CjcC7IQ!KEyyMCdUwDVR4bHWSotIUaQMmv2K_8VcuH53rmagNr1NcYEEz-_Luy3S7RU-CByIo_dYzQZihrabsA9qHXZKnQ$). | 0 |
| 83 | 80 not (81 or 82) | 53 |
| 84 | limit 83 to english language | 52 |
| 85 | 83 not 84 | 1 |
| 86 | (2021-07-06 or 2021-07-07 or 2021-07-08 or 2021-07-09 or 2021-07-1* or 2021-07-2* or 2021-07-3* or 2021-08* or 2021-09* or 2021-1* or 2022* or 2023* or 2024* or 2025*).dl. | 486,120 |
| 87 | 84 and 86 | 19 |
| 88 | 85 and 86 | 0 |

*******************************

**Database:** APA PsycInfo <1806 to January 2025 Week 1>

| **#** | **Query** | **Results from 10 Jan 2025** |
| --- | --- | --- |
| 1 | exp Correctional Institutions/ | 12,451 |
| 2 | exp Prisoners/ | 13,627 |
| 3 | Criminal Offenders/ | 16,287 |
| 4 | exp Criminal Justice/ or Criminal Law/ | 18,146 |
| 5 | Legal Processes/ or probation/ or Parole/ | 18,528 |
| 6 | (jurisprudenc* or ligitat*).ti,ab. | 1,348 |
| 7 | (legal adj (system? or servic*)).ti,ab. | 4,734 |
| 8 | (prisoner* or prison? or imprison*).ti,ab. | 29,616 |
| 9 | (inmate* or convict* or criminal* or offender?).ti,ab. | 91,542 |
| 10 | (correctional adj2 (setting? or service? or units or unit or facility or facilities or institution* or centre* or center*)).ti,ab. | 4,697 |
| 11 | (penal adj2 (setting? or service? or units or unit or facility or facilities or institution* or centre* or center*)).ti,ab. | 446 |
| 12 | (jail* or penitentiar* or gaol*).ti,ab. | 5,535 |
| 13 | incarcerat*.ti,ab. | 16,516 |
| 14 | (detain* or detention?).ti,ab. | 6,915 |
| 15 | parole?.ti,ab. | 3,088 |
| 16 | probation*.ti,ab. | 5,508 |
| 17 | felon*.ti,ab. | 1,758 |
| 18 | Police Personnel/ or exp Law Enforcement/ | 55,849 |
| 19 | (police or policing).ti,ab. | 29,812 |
| 20 | law enforce*.ti,ab. | 9,404 |
| 21 | forensic*.ti,ab. | 21,668 |
| 22 | Forensic Psychiatry/ or Forensic Psychology/ | 10,780 |
| 23 | (correctional or forensic).jx. | 6,672 |
| 24 | or/1-23 | 188,593 |
| 25 | exp rehabilitation/ | 64,123 |
| 26 | rehab*.ti,ab,jx. | 93,875 |
| 27 | telerehab*.ti,ab,jx. | 350 |
| 28 | neurorehab*.ti,ab,jx. | 5,383 |
| 29 | (physiatrist? or physiatry).ti,ab. | 165 |
| 30 | exp Rehabilitation Centers/ | 1,287 |
| 31 | occupational therapy/ | 7,901 |
| 32 | (occupational adj therap*).ti,ab,jx. | 16,863 |
| 33 | Physical therapy/ | 3,971 |
| 34 | (physical adj therap*).ti,ab,jx. | 4,647 |
| 35 | physiotherap*.ti,ab,jx. | 4,371 |
| 36 | physio-therapist*.ti,ab,jx. | 0 |
| 37 | Speech Therapists/ | 1,944 |
| 38 | (speech adj2 (therap* or patholog*)).ti,ab,jx. | 11,999 |
| 39 | Neuropsychology/ | 22,150 |
| 40 | Neuropsycholog*.ti,ab,jx. | 90,419 |
| 41 | (Nutritionist? or Dietician?).ti,ab,jx. | 910 |
| 42 | (therap* adj recreation*).ti,ab,jx. | 606 |
| 43 | child life specialist?.ti,ab. | 142 |
| 44 | play therapy/ | 4,442 |
| 45 | (play adj therap*).ti,ab. | 3,715 |
| 46 | Respite Care/ | 516 |
| 47 | respite.ti,ab. | 1,983 |
| 48 | exp Social Casework/ | 23,076 |
| 49 | Case Management/ | 3,720 |
| 50 | case manag*.ti,ab. | 7,644 |
| 51 | exp Social Workers/ | 16,095 |
| 52 | social work*.ti,ab,jx. | 68,773 |
| 53 | (nurse? or nursing).ti,ab,jx. | 149,952 |
| 54 | Reintegration/ or exp Social Integration/ | 9,937 |
| 55 | (integrat* or reintegrat* or re-integrat* or reentry or re-entry or resettle* or re-settle*).ti,ab. | 294,617 |
| 56 | Aftercare/ | 1,238 |
| 57 | (Aftercare or "after care").ti,ab. | 3,196 |
| 58 | "transitional care".ti,ab. | 486 |
| 59 | or/25-58 | 720,034 |
| 60 | exp Brain Injuries/ | 26,391 |
| 61 | exp traumatic brain injury/ | 24,772 |
| 62 | Brain concussion/ | 3,860 |
| 63 | head injuries/ | 4,912 |
| 64 | tbi*2.ti,ab. | 13,940 |
| 65 | mtbi*2.ti,ab. | 2,687 |
| 66 | concuss*.ti,ab. | 4,621 |
| 67 | postconcuss*.ti,ab. | 974 |
| 68 | ((head* or brain* or cerebr* or crani* or skull* or intracran*) adj2 (injur* or trauma* or damag* or wound* or swell* or oedema* or edema* or fracture* or contusion* or pressur*)).ti,ab,jx. | 56,575 |
| 69 | ((brain* or cerebr* or intracerebr* or crani* or intracran* or head* or subdural* or epidural* or extradural*) adj (haematoma* or hematoma* or hemorrhag* or haemorrhag* or bleed*)).ti,ab. | 3,985 |
| 70 | Cognitive Impairment/ or Mild Cognitive Impairment/ | 51,785 |
| 71 | ((cogniti* or neurocogniti*) adj2 (impair* or dysfunction* or disorder* or declin*)).ti,ab. | 84,970 |
| 72 | or/60-71 | 156,418 |
| 73 | 24 and 59 and 72 | 1,000 |
| 74 | limit 73 to ("column/opinion" or dissertation or editorial) | 77 |
| 75 | 73 not 74 | 923 |
| 76 | limit 75 to animal | 3 |
| 77 | limit 75 to human | 894 |
| 78 | 75 not (76 not 77) | 923 |
| 79 | limit 78 to english language | 882 |
| 80 | 78 not 79 | 41 |
| 81 | limit 79 to up=20210706-20250110 | 87 |
| 82 | limit 80 to up=20210706-20250110 | 12 |

***************************

**Database:** CINAHL Ultimate

Interface: EBSCOhost

Date Searched: January 10, 2025

| **#** | **Query** | **Limiters/Expanders** | **Results** |
| --- | --- | --- | --- |
| S1 | (MH "Correctional Facilities") | Expanders - Apply equivalent subjects Search modes - Proximity | 7,524 |
| S2 | (MH "Prisoners") | Expanders - Apply equivalent subjects Search modes - Proximity | 10,720 |
| S3 | (MH "Public Offenders+") | Expanders - Apply equivalent subjects Search modes - Proximity | 12,454 |
| S4 | (MH "Jurisprudence") OR (MH "Criminal Justice") | Expanders - Apply equivalent subjects Search modes - Proximity | 9,297 |
| S5 | TI ( (jurisprudenc* or ligitat*) ) OR AB ( (jurisprudenc* or ligitat*) ) | Expanders - Apply equivalent subjects Search modes - Proximity | 449 |
| S6 | TI ( (legal n1 (system* or servic*)) ) OR AB ( (legal n1 (system* or servic*)) ) | Expanders - Apply equivalent subjects Search modes - Proximity | 1,693 |
| S7 | TI ( (prison* or imprison*) ) OR AB ( (prison* or imprison*) ) | Expanders - Apply equivalent subjects Search modes - Proximity | 10,640 |
| S8 | TI ( (inmate* or convict* or criminal* or offender*) ) OR AB ( (inmate* or convict* or criminal* or offender*) ) | Expanders - Apply equivalent subjects Search modes - Proximity | 22,083 |
| S9 | TI ( (correctional n2 (setting or settings or service or services or units or unit or facility or facilities or institution* or centre* or center*)) ) OR AB ( (correctional n2 (setting or settings or service or services or units or unit or facility or facilities or institution* or centre* or center*)) ) | Expanders - Apply equivalent subjects Search modes - Proximity | 1,739 |
| S10 | TI ( (penal n2 (setting or settings or service or services or units or unit or facility or facilities or institution* or centre* or center*)) ) OR AB ( (penal n2 (setting or settings or service or services or units or unit or facility or facilities or institution* or centre* or center*)) ) | Expanders - Apply equivalent subjects Search modes - Proximity | 57 |
| S11 | TI ( (jail* or penitentiar* or gaol*) ) OR AB ( (jail* or penitentiar* or gaol*) ) | Expanders - Apply equivalent subjects Search modes - Proximity | 2,851 |
| S12 | TI incarcerat* OR AB incarcerat* | Expanders - Apply equivalent subjects Search modes - Proximity | 7,513 |
| S13 | TI ( detain* or detention* ) OR AB ( detain* or detention* ) | Expanders - Apply equivalent subjects Search modes - Proximity | 3,193 |
| S14 | TI parole* OR AB parole* | Expanders - Apply equivalent subjects Search modes - Proximity | 665 |
| S15 | TI probation* OR AB probation* | Expanders - Apply equivalent subjects Search modes - Proximity | 1,186 |
| S16 | TI felon* OR AB felon* | Expanders - Apply equivalent subjects Search modes - Proximity | 361 |
| S17 | TI ( police or policing ) OR AB ( police or policing ) | Expanders - Apply equivalent subjects Search modes - Proximity | 10,206 |
| S18 | TI law enforce* OR AB law enforce* | Expanders - Apply equivalent subjects Search modes - Proximity | 3,145 |
| S19 | TI forensic* OR AB forensic* | Expanders - Apply equivalent subjects Search modes - Proximity | 12,312 |
| S20 | (MH "Police") | Expanders - Apply equivalent subjects Search modes - Proximity | 7,557 |
| S21 | (MH "Forensic Psychiatry+") | Expanders - Apply equivalent subjects Search modes - Proximity | 2,156 |
| S22 | SO (correctional or forensic) | Expanders - Apply equivalent subjects Search modes - Proximity | 11,189 |
| S23 | S1 OR S2 OR S3 OR S4 OR S5 OR S6 OR S7 OR S8 OR S9 OR S10 OR S11 OR S12 OR S13 OR S14 OR S15 OR S16 OR S17 OR S18 OR S19 OR S20 OR S21 OR S22 | Expanders - Apply equivalent subjects Search modes - Proximity | 83,652 |
| S24 | (MH "Rehabilitation+") | Expanders - Apply equivalent subjects Search modes - Proximity | 354,727 |
| S25 | (MH "Physical Medicine") | Expanders - Apply equivalent subjects Search modes - Proximity | 2,121 |
| S26 | TI rehab* OR AB rehab* OR SO rehab* | Expanders - Apply equivalent subjects Search modes - Proximity | 193,174 |
| S27 | TI telerehab* OR AB telerehab* OR SO telerehab* | Expanders - Apply equivalent subjects Search modes - Proximity | 1,132 |
| S28 | TI neurorehab* OR AB neurorehab* OR SO neurorehab* | Expanders - Apply equivalent subjects Search modes - Proximity | 6,887 |
| S29 | TI ( (physiatrist* or physiatry) ) OR AB ( (physiatrist* or physiatry) ) | Expanders - Apply equivalent subjects Search modes - Proximity | 1,203 |
| S30 | (MH "Rehabilitation Centers+") | Expanders - Apply equivalent subjects Search modes - Proximity | 10,061 |
| S31 | (MH "Occupational Therapy+") or (MH "Occupational Therapists") | Expanders - Apply equivalent subjects Search modes - Proximity | 38,398 |
| S32 | TI (occupational n1 therap*) OR AB (occupational n1 therap*) OR SO (occupational n1 therap*) | Expanders - Apply equivalent subjects Search modes - Proximity | 60,903 |
| S33 | (MH "Physical Therapy+") OR (MH "Physical Therapists") | Expanders - Apply equivalent subjects Search modes - Proximity | 179,921 |
| S34 | TI (physical n1 therap*) OR AB (physical n1 therap*) OR SO (physical n1 therap*) | Expanders - Apply equivalent subjects Search modes - Proximity | 54,400 |
| S35 | TI physiotherap* OR AB physiotherap* OR SO physiotherap* | Expanders - Apply equivalent subjects Search modes - Proximity | 50,701 |
| S36 | TI physio-therapist* OR AB physio-therapist* OR SO physio-therapist* | Expanders - Apply equivalent subjects Search modes - Proximity | 9 |
| S37 | (MH "Speech-Language Pathologists") OR (MH "Speech-Language Pathology Assistants") | Expanders - Apply equivalent subjects Search modes - Proximity | 8,736 |
| S38 | TI ( (speech n2 (therap* or patholog*)) ) OR AB ( (speech n2 (therap* or patholog*)) ) OR SO ( (speech n2 (therap* or patholog*)) ) | Expanders - Apply equivalent subjects Search modes - Proximity | 18,711 |
| S39 | (MH "Neuropsychology") | Expanders - Apply equivalent subjects Search modes - Proximity | 2,286 |
| S40 | TI Neuropsycholog* OR AB Neuropsycholog* OR SO Neuropsycholog | Expanders - Apply equivalent subjects Search modes - Proximity | 17,312 |
| S41 | (MH "Dietitians") | Expanders - Apply equivalent subjects Search modes - Proximity | 6,405 |
| S42 | TI ( (Nutritionist* or Dietician*) ) OR AB ( (Nutritionist* or Dietician*) ) OR SO ( (Nutritionist* or Dietician*) ) | Expanders - Apply equivalent subjects Search modes - Proximity | 3,081 |
| S43 | (MH "Recreational Therapy") | Expanders - Apply equivalent subjects Search modes - Proximity | 1,847 |
| S44 | (MH "Recreational Therapists") | Expanders - Apply equivalent subjects Search modes - Proximity | 224 |
| S45 | TI (therap* n1 recreation*) OR AB (therap* n1 recreation*) OR SO (therap* n1 recreation*) | Expanders - Apply equivalent subjects Search modes - Proximity | 1,701 |
| S46 | TI child life specialist* OR AB child life specialist* OR SO child life specialist* | Expanders - Apply equivalent subjects Search modes - Proximity | 192 |
| S47 | (MH "Play Therapy") | Expanders - Apply equivalent subjects Search modes - Proximity | 1,531 |
| S48 | TI (play n1 therap*) OR AB (play n1 therap*) OR (play n1 therap*) | Expanders - Apply equivalent subjects Search modes - Proximity | 2,828 |
| S49 | (MH "Respite Care") | Expanders - Apply equivalent subjects Search modes - Proximity | 1,518 |
| S50 | TI respite OR AB respite | Expanders - Apply equivalent subjects Search modes - Proximity | 2,038 |
| S51 | (MH "Case Managers") | Expanders - Apply equivalent subjects Search modes - Proximity | 4,664 |
| S52 | (MH "Case Management") | Expanders - Apply equivalent subjects Search modes - Proximity | 18,386 |
| S53 | TI "case manag*" OR AB "case manag*" | Expanders - Apply equivalent subjects Search modes - Proximity | 12,940 |
| S54 | (MH "Social Work+") | Expanders - Apply equivalent subjects Search modes - Proximity | 16,558 |
| S55 | (MH "Social Workers") | Expanders - Apply equivalent subjects Search modes - Proximity | 12,663 |
| S56 | TI "social work*" OR AB "social work*" OR SO "social work*" | Expanders - Apply equivalent subjects Search modes - Proximity | 55,916 |
| S57 | (MH "Forensic Nursing") | Expanders - Apply equivalent subjects Search modes - Proximity | 1,840 |
| S58 | (MH "Correctional Health Nursing") | Expanders - Apply equivalent subjects Search modes - Proximity | 7,382 |
| S59 | TI ( (nurse or nurses or nursing) ) OR AB ( (nurse or nurses or nursing) ) OR SO ( (nurse or nurses or nursing) ) | Expanders - Apply equivalent subjects Search modes - Proximity | 1,361,577 |
| S60 | (MH "Community Reintegration") | Expanders - Apply equivalent subjects Search modes - Proximity | 2,313 |
| S61 | TI ( (integrat* or reintegrat* or re-integrat* or reentry or re-entry or resettle* or re-settle) ) OR AB ( (integrat* or reintegrat* or re-integrat* or reentry or re-entry or resettle* or re-settle) ) | Expanders - Apply equivalent subjects Search modes - Proximity | 180,485 |
| S62 | (MH "After Care") | Expanders - Apply equivalent subjects Search modes - Proximity | 22,845 |
| S63 | TI ( (Aftercare or "after care") ) OR AB ( (Aftercare or "after care") ) | Expanders - Apply equivalent subjects Search modes - Proximity | 1,911 |
| S64 | TI "transitional care" OR AB "transitional care" | Expanders - Apply equivalent subjects Search modes - Proximity | 1,763 |
| S65 | (MH "Transitional Care") | Expanders - Apply equivalent subjects Search modes - Proximity | 4,571 |
| S66 | S24 OR S25 OR S26 OR S27 OR S28 OR S29 OR S30 OR S31 OR S32 OR S33 OR S34 OR S35 OR S36 OR S37 OR S38 OR S39 OR S40 OR S41 OR S42 OR S43 OR S44 OR S45 OR S46 OR S47 OR S48 OR S49 OR S50 OR S51 OR S52 OR S53 OR S54 OR S55 OR S56 OR S57 OR S58 OR S59 OR S60 OR S61 OR S62 OR S63 OR S64 OR S65 | Expanders - Apply equivalent subjects Search modes - Proximity | 2,134,795 |
| S67 | (MH "Brain Injuries+") | Expanders - Apply equivalent subjects Search modes - Proximity | 34,790 |
| S68 | (MH "Head Injuries") | Expanders - Apply equivalent subjects Search modes - Proximity | 8,352 |
| S69 | (MH "Brain Concussion+") | Expanders - Apply equivalent subjects Search modes - Proximity | 7,014 |
| S70 | TI ( TBI* OR mTBI* ) OR AB ( TBI* OR mTBI* ) | Expanders - Apply equivalent subjects Search modes - Proximity | 12,164 |
| S71 | TI ( concuss* or postconcuss* ) OR AB ( concuss* or postconcuss* ) | Expanders - Apply equivalent subjects Search modes - Proximity | 7,456 |
| S72 | TI ( ((head* or brain* or cerebr* or crani* or skull* or intracran*) n2 (injur* or trauma* or damag* or wound* or swell* or oedema* or edema* or fracture* or contusion* or pressur*)) ) OR AB ( ((head* or brain* or cerebr* or crani* or skull* or intracran*) n2 (injur* or trauma* or damag* or wound* or swell* or oedema* or edema* or fracture* or contusion* or pressur*)) ) OR SO ( ((head* or brain* or cerebr* or crani* or skull* or intracran*) n2 (injur* or trauma* or damag* or wound* or swell* or oedema* or edema* or fracture* or contusion* or pressur*)) ) | Expanders - Apply equivalent subjects Search modes - Proximity | 59,499 |
| S73 | TI ( ((brain* or cerebr* or intracerebr* or crani* or intracran* or head* or subdural* or epidural* or extradural*) n1 (haematoma* or hematoma* or hemorrhag* or haemorrhag* or bleed*)) ) OR AB ( ((brain* or cerebr* or intracerebr* or crani* or intracran* or head* or subdural* or epidural* or extradural*) n1 (haematoma* or hematoma* or hemorrhag* or haemorrhag* or bleed*)) ) | Expanders - Apply equivalent subjects Search modes - Proximity | 15,825 |
| S74 | (MH "Cognition Disorders+") | Expanders - Apply equivalent subjects Search modes - Proximity | 40,250 |
| S75 | TI ( ((cogniti* or neurocogniti*) n2 (impair* or dysfunction* or disorder* or declin*)) ) OR AB ( ((cogniti* or neurocogniti*) n2 (impair* or dysfunction* or disorder* or declin*)) ) | Expanders - Apply equivalent subjects Search modes - Proximity | 57,621 |
| S76 | S67 OR S68 OR S69 OR S70 OR S71 OR S72 OR S73 OR S74 OR S75 | Expanders - Apply equivalent subjects Search modes - Proximity | 160,321 |
| S77 | S23 AND S66 AND S76 | Expanders - Apply equivalent subjects Search modes - Proximity | 406 |
| S78 | S77 | Limiters - English Language Expanders - Apply equivalent subjects Search modes - Proximity | 402 |
| S79 | S77 NOT S78 | Expanders - Apply equivalent subjects Search modes - Proximity | 4 |
| S80 | (EM 20210706- OR (ZD "in process" AND RD 20210706-)) | Expanders - Apply equivalent subjects Search modes - Proximity | 1,837,341 |
| S81 | S78 AND S80 | Expanders - Apply equivalent subjects Search modes - Proximity | 60 |
| S82 | S79 AND S80 | Expanders - Apply equivalent subjects Search modes - Proximity | 0 |

*************************

**Database:** Criminal Justice Abstracts

Interface: EBSCOhost

Search Date: January 10, 2025

| **#** | **Query** | **Limiters/Expanders** | **Results** |
| --- | --- | --- | --- |
| S1 | ( TI rehab* OR AB rehab* OR SO rehab* ) OR ( TI neurorehab* OR AB neurorehab* OR SO neurorehab* ) OR ( TI telerehab* OR AB telerehab* OR SO telerehab* ) OR ( TI ( (physiatrist* or physiatry) ) OR AB ( (physiatrist* or physiatry) ) ) | Expanders - Apply equivalent subjects Search modes - Proximity | 10,763 |
| S2 | ( TI (occupational n1 therap*) OR AB (occupational n1 therap*) OR SO (occupational n1 therap*) ) OR ( TI (physical n1 therap*) OR AB (physical n1 therap*) OR SO (physical n1 therap*) ) OR ( TI physiotherap* OR AB physiotherap* OR SO physiotherap* TI physio-therapist* OR AB physio-therapist* OR SO physio-therapist* ) OR ( TI ( (speech n2 (therap* or patholog*)) ) OR AB ( (speech n2 (therap* or patholog*)) ) OR SO ( (speech n2 (therap* or patholog*)) ) ) OR ( TI Neuropsycholog* OR AB Neuropsycholog* OR SO Neuropsycholog ) OR ( TI ( (Nutritionist* or Dietician*) ) OR AB ( (Nutritionist* or Dietician*) ) OR SO ( (Nutritionist* or Dietician*) ) ) OR ( TI (therap* n1 recreation*) OR AB (therap* n1 recreation*) OR SO (therap* n1 recreation*) ) OR ( TI child life specialist* OR AB child life specialist* OR SO child life specialist* ) OR ( TI (play n1 therap*) OR AB (play n1 therap*) OR (play n1 therap*) ) OR ( TI respite OR AB respite ) OR ( TI "case manag*" OR AB "case manag*" ) OR ( TI "social work*" OR AB "social work*" OR SO "social work*" ) | Expanders - Apply equivalent subjects Search modes - Proximity | 24,641 |
| S3 | ( TI ( (nurse or nurses or nursing) ) OR AB ( (nurse or nurses or nursing) ) OR SO ( (nurse or nurses or nursing) ) ) OR ( TI ( (integrat* or reintegrat* or re-integrat* or reentry or re-entry or resettle* or re-settle) ) OR AB ( (integrat* or reintegrat* or re-integrat* or reentry or re-entry or resettle* or re-settle) ) ) OR ( TI ( (Aftercare or "after care") ) OR AB ( (Aftercare or "after care") ) ) OR ( TI "transitional care" OR AB "transitional care" ) | Expanders - Apply equivalent subjects Search modes - Proximity | 30,163 |
| S4 | S1 OR S2 OR S3 | Expanders - Apply equivalent subjects Search modes - Proximity | 61,978 |
| S5 | ( TI ( TBI* OR mTBI* ) OR AB ( TBI* OR mTBI* ) ) OR ( TI ( concuss* or postconcuss* ) OR AB ( concuss* or postconcuss* ) ) OR ( TI ( ((head* or brain* or cerebr* or crani* or skull* or intracran*) n2 (injur* or trauma* or damag* or wound* or swell* or oedema* or edema* or fracture* or contusion* or pressur*)) ) OR AB ( ((head* or brain* or cerebr* or crani* or skull* or intracran*) n2 (injur* or trauma* or damag* or wound* or swell* or oedema* or edema* or fracture* or contusion* or pressur*)) ) OR SO ( ((head* or brain* or cerebr* or crani* or skull* or intracran*) n2 (injur* or trauma* or damag* or wound* or swell* or oedema* or edema* or fracture* or contusion* or pressur*)) ) ) OR ( TI ( ((brain* or cerebr* or intracerebr* or crani* or intracran* or head* or subdural* or epidural* or extradural*) n1 (haematoma* or hematoma* or hemorrhag* or haemorrhag* or bleed*)) ) OR AB ( ((brain* or cerebr* or intracerebr* or crani* or intracran* or head* or subdural* or epidural* or extradural*) n1 (haematoma* or hematoma* or hemorrhag* or haemorrhag* or bleed*)) ) ) OR ( TI ( ((cogniti* or neurocogniti*) n2 (impair* or dysfunction* or disorder* or declin*)) ) OR AB ( ((cogniti* or neurocogniti*) n2 (impair* or dysfunction* or disorder* or declin*)) ) ) | Expanders - Apply equivalent subjects Search modes - Proximity | 3,942 |
| S6 | S4 AND S5 | Expanders - Apply equivalent subjects Search modes - Proximity | 467 |
| S7 | S6 | Limiters - Language: English Expanders - Apply equivalent subjects Search modes - Proximity | 463 |
| S8 | S6 NOT S7 | Expanders - Apply equivalent subjects Search modes - Proximity | 4 |
| S9 | ZD (20210706 OR 20210707 OR 20210708 OR 20210709 OR 2021071* OR 2021072* OR 2021073* OR 202108* OR 202109* OR 20211* OR 2022* OR 2023* OR 2024* OR 2025*) | Expanders - Apply equivalent subjects Search modes - Proximity | 95,397 |
| S10 | S7 AND S9 | Expanders - Apply equivalent subjects Search modes - Proximity | 70 |
| S11 | S8 AND S9 | Expanders - Apply equivalent subjects Search modes - Proximity | 3 |

*********************

**Database:** Nursing and Allied Health Premium

Interface: ProQuest

Search Date: January 10, 2025

(NOFT(jurisprudenc* OR ligitat*) OR NOFT(legal n/1 system*) OR NOFT(legal n/1 servic*) OR NOFT(prison* OR imprison* OR inmate* OR convict* OR criminal* OR offender* OR jail* OR penitentiar* OR gaol*) OR NOFT(correctional n/2 (setting or settings or service or services or units or unit or facility or facilities or institution* or centre* or center*)) OR NOFT(penal n2 (setting OR settings OR service OR services OR units OR unit OR facility OR facilities OR institution* OR centre* OR center*)) OR NOFT(incarcerat* OR detain* OR detention* OR parole* OR probation* OR police OR policing OR forensic*) OR NOFT("law enforce*") OR JN(correctional OR forensic)) AND (NOFT(TBI* OR mTBI* OR concuss* OR postconcuss*) OR NOFT((head* or brain* or cerebr* or crani* or skull* or intracran*) n/2 (injur* or trauma* or damag* or wound* or swell* or oedema* or edema* or fracture* or contusion* or pressur*)) OR PUB((head* or brain* or cerebr* or crani* or skull* or intracran*) n/2 (injur* or trauma* or damag* or wound* or swell* or oedema* or edema* or fracture* or contusion* or pressur*)) OR NOFT((brain* or cerebr* or intracerebr* or crani* or intracran* or head* or subdural* or epidural* or extradural*) n/1 (haematoma* or hematoma* or hemorrhag* or haemorrhag* or bleed*)) OR NOFT((cogniti* or neurocogniti*) n/2 (impair* or dysfunction* or disorder* or declin*))) AND (NOFT(rehab* OR telerehab* OR neurorehab* OR physiatrist* OR physiatry OR Neuropsycholog* OR Nutritionist* or Dietician* OR respite) OR PUB(rehab* OR telerehab* OR neurorehab* OR Neuropsycholog* OR Nutritionist* or Dietician* OR Respite) OR NOFT(occupational n/1 therap*) OR PUB(occupational n/1 therap*) OR NOFT(physical n1 therap*) OR PUB(physical n/1 therap*) OR NOFT(physiotherap* or "physio-therap*") OR PUB(physiotherap* or "physio-therap*") OR NOFT(speech n/2 (therap* or patholog*)) OR PUB(speech n/2 (therap* or patholog*)) OR NOFT(therap* n/1 recreation*) OR PUB(therap* n/1 recreation*) OR NOFT("child life specialist*") OR NOFT(play n/1 therap*) OR NOFT("case manag*" ) OR NOFT("social work*" ) OR NOFT(nurse or nurses or nursing) Or NOFT(integrat* or reintegrat* or "re-integrat*" or reentry or "re-entry" or resettle* or "re-settle") OR NOFT(Aftercare or "after care") OR NOFT("transitional care"))

TOTAL: 271 Results (270 English, 1 Other Languages)
Updated Results Only: 83 English, 1 Other Languages

**********************

**Database:** Applied Social Sciences Index & Abstracts (ASSIA)

Interface: ProQuest

Search Date: January 10, 2025

(NOFT(jurisprudenc* OR ligitat*) OR NOFT(legal n/1 system*) OR NOFT(legal n/1 servic*) OR NOFT(prison* OR imprison* OR inmate* OR convict* OR criminal* OR offender* OR jail* OR penitentiar* OR gaol*) OR NOFT(correctional n/2 (setting or settings or service or services or units or unit or facility or facilities or institution* or centre* or center*)) OR NOFT(penal n2 (setting OR settings OR service OR services OR units OR unit OR facility OR facilities OR institution* OR centre* OR center*)) OR NOFT(incarcerat* OR detain* OR detention* OR parole* OR probation* OR police OR policing OR forensic*) OR NOFT("law enforce*") OR JN(correctional OR forensic)) AND (NOFT(TBI* OR mTBI* OR concuss* OR postconcuss*) OR NOFT((head* or brain* or cerebr* or crani* or skull* or intracran*) n/2 (injur* or trauma* or damag* or wound* or swell* or oedema* or edema* or fracture* or contusion* or pressur*)) OR PUB((head* or brain* or cerebr* or crani* or skull* or intracran*) n/2 (injur* or trauma* or damag* or wound* or swell* or oedema* or edema* or fracture* or contusion* or pressur*)) OR NOFT((brain* or cerebr* or intracerebr* or crani* or intracran* or head* or subdural* or epidural* or extradural*) n/1 (haematoma* or hematoma* or hemorrhag* or haemorrhag* or bleed*)) OR NOFT((cogniti* or neurocogniti*) n/2 (impair* or dysfunction* or disorder* or declin*))) AND (NOFT(rehab* OR telerehab* OR neurorehab* OR physiatrist* OR physiatry OR Neuropsycholog* OR Nutritionist* or Dietician* OR respite) OR PUB(rehab* OR telerehab* OR neurorehab* OR Neuropsycholog* OR Nutritionist* or Dietician* OR Respite) OR NOFT(occupational n/1 therap*) OR PUB(occupational n/1 therap*) OR NOFT(physical n1 therap*) OR PUB(physical n/1 therap*) OR NOFT(physiotherap* or "physio-therap*") OR PUB(physiotherap* or "physio-therap*") OR NOFT(speech n/2 (therap* or patholog*)) OR PUB(speech n/2 (therap* or patholog*)) OR NOFT(therap* n/1 recreation*) OR PUB(therap* n/1 recreation*) OR NOFT("child life specialist*") OR NOFT(play n/1 therap*) OR NOFT("case manag*" ) OR NOFT("social work*" ) OR NOFT(nurse or nurses or nursing) Or NOFT(integrat* or reintegrat* or "re-integrat*" or reentry or "re-entry" or resettle* or "re-settle") OR NOFT(Aftercare or "after care") OR NOFT("transitional care"))

TOTAL: 170 Results (170 English, 0 Other Languages)

Updated Results Only: 44 English, 0 Other Languages
