# Supplementary material for: Exploring compassionate care in rehabilitation among individuals who are involved with the criminal-legal system with traumatic brain injury: A scoping review
Source: PLoS One. 2026 Jun 24;21(6):e0341381. doi: 10.1371/journal.pone.0341381 (PMC13293406; doi:10.1371/journal.pone.0341381)
Supplement: S2 File — BISI: Brain Injury Screening Index; CHAT: Comprehensive Health Assessment Tool; DUI: Driving Under the Influence; GAD-7: Generalized Anxiety Disorder-7; M2PI: Mayo-Portland Adaptability Inventory-4 Participation Index; MPAI-4: Mayo-Portland Adaptability Inventory-4; NRP: Neurobehavioural Resource Project; PHQ-9: Patient Health Questionnaire-9; RF: Resource Facilitation. (DOCX) [file pone.0341381.s002.docx]

**S2a Table. Charting Table of Compassionate Care.**

| **Study (Author, Year)** | **Country** | **Awareness of Patient Suffering** | **Judgement and Consideration** | **Motivation to Alleviate Patient Suffering** |
| --- | --- | --- | --- | --- |
| Ahlers et al., 2024 | UK | Use of screening tools (e.g., CHAT)  Assessment of health needs, TBI, impairments, developmental history, comorbid needs  Neurocognitive assessments | Develop personalized goals, develop summary reports, and identify recommendations  Discharge and community intervention planning | Education on TBI, behavioural strategies, copies strategies, care plan, referral to specialist brain injury service (if required)  Ongoing supports 8-weeks post release (e.g., helping patient organize and attend appointments) |
| Boglo et al., 2023 | UK | Screening tools (e.g., BISI)  Identification of history  Questionnaires and standardized assessment to explore cognitive function, mood, mental health | Developing adaptive ways to cope with difficulties (e.g., psychoeducation, compensatory strategies, therapy)  Setting of personal goals | Establishment of relationships with relevant agencies and care providers (e.g., employment, housing, physiotherapist, social worker, probation officer)  Assessment and intervention reports (letters to agencies, general practitioner, next of kin, etc.) |
| Chitsabesan et al., 2015 | USA | Awareness that substance abuse and mental health problems may be comorbid  Awareness of the impacts of outcomes and ability for long-term rehabilitation these comorbid conditions may have  Staff apprenticeship program for NRP staff | Considered the "Ten Principles Governing Delivery of Services and Supports  Within the Neurobehavioural Resource Project" when planning intervention  Person-centred service to help dictate their goals, procedures to achieve goals, amount of support, etc.  Interventions and supports are organized around personally meaningful activities  Components of life must be integrated | Establish positive everyday routines  Ongoing assessment of progress and alleviation of supports as needed  Provide behavioural supports that are positive and proactive |
| Ramos et al., 2018 | USA | HELPS Brain Injury Screening Tool, Traumatic Brain Injury Screening tool + screened for "behavioural health diagnoses", substance use disorder, and trauma history  Specialized to work with individuals with brain injuries | Administered M2PI and MPAI-4 to assess the efficacies of case management  Case managers provided services in person or over the phone | Case management/ resource facilitation  Individual and group psychotherapy  Group substance abuse therapy  DUI therapy and education  Medication management and employment services |
| Trexler & Parrott, 2022 | UK | Identifying needs through clinical interviews and screening tools (e.g., BISI, GAD-7, PHQ-9)  Review medical notes and offence history  Staff education | Setting of personal goals  Formation of person-centred intervention plan  Liaising with family and friends  Written behavioural support guidelines | Brain injury awareness training to correctional staff  Linking with specific services to provide support where needed  Assessment and intervention reports, letters to probation officers, agencies, general practitioners, etc. (as required)  Follow-up after release through consulting with probation delivery units for person management |
| Ylvisaker et al., 2007 | USA | **RF:** 8-hour evaluation of cognitive and neurobehavioural functions, substance abuse, family and social support, etc.  **Modified RF:** Evaluation of cognitive/ neurocognitive functions, substance abuse, and level of disability | Case conferences  Identification of needs and assessment of services available to meet needs | **RF:** monthly RF team conferences to assess progress, vocational placement services for 90-days, contact every 2 weeks for 12 months  TBI education to patient, family, and assisting community service providers (e.g., employer)  **Modified RF:** TBI education to patient and family, navigation to multiple community services and supports, weekly TBI Education and Life Skills Groups  Creation of TBI wallet cards containing information about patient's injury and summary of evaluation for each client |
| **BISI:** Brain Injury Screening Index; **CHAT:** Comprehensive Health Assessment Tool; **DUI:** Driving Under the Influence; **GAD-7:** Generalized Anxiety Disorder- 7; **HELPS:** Hit Head/ Been Hit on the Head, Emergency room, Loss of consciousness, Problems in daily life, Significant Sickness; **M2PI:** Mayo-Portland Adaptability Inventory- 4 Participation Index; **MPAI-4:** Mayo-Portland Adaptability Inventory- 4; **NRP:** Neurobehavioural Resource Project; **PHQ-9:** Patient Health Questionnaire- 9; **RF:** Resource Facilitation | | | | |

**S2b Table. Charting Table of Newly Identified Articles from Updated Search.**

| **Study (Author, Year)** | **Country** | **Study Design and Objectives** | **Study Sample** | **TBI Status** | **Criminal Justice Status** | **Rehabilitation Intervention, Team, Outcome** | **TBI-Specific Facilitators, Barriers, Gaps** |
| --- | --- | --- | --- | --- | --- | --- | --- |
| Ahlers et al., 2024 | USA | Cohort Study  To examine the effectiveness of case management services for a population of justice-involved individuals with TBI history | **Study 1 (N = 485):**  Mean age [Years]: 36 (SD = 11.9), 18-80 years old  Male: 68.9%  Female: 31.1%  TBI Positive: 36.4%  TBI Negative: 63.6%  **Study 2 (N = 25):**  Age (N= 20)  - 20-29: 16%  - 30-39: 12%  - 40-49: 28%  - 50-59: 16%  - 60-69: 8%  Gender (N = 20)  - Male: 60%  - Female: 20%  Education (N = 19)  - 8th grade: 4%  - High school: 44%  - Some college: 16%  - Bachelor's degree: 8%  - Master’s degree: 0%  - Doctorate/ Post Master's: 4%  Ethnicity (N = 20)  - American Indian: 0.5%  - Hispanic: 33.0%  - White: 57.1% | **Study 1**  Screening:  - HELPS Brain Injury Screening Tool  - Traumatic Brain Injury Screening Tool  - Screened for "behavioural health diagnoses"  - Trauma history  - Substance use disorders | **Intersection**  - Parole  6-month post-release from correctional facility | **Intervention**  Study 1: Jail-Based Behavioural Health System Program  - Case management/ resource facilitation  - Individual and group psychotherapy  - Group substance abuse therapy  - DUI therapy and education  - Medication management and employment  Study 2: Brain Injury Alliance of Colorado  - Conducted case management in person or over the phone  - Administered the M2PI and MPAI-4 at beginning and 6-month mark to measure engagement after brain injury and success of case management  **Rehabilitation Team**  - Case managers  **Outcomes**  - Improve quality of life  - Reducing rearrest rates  - Promoting community engagement  **Funding of Rehabilitation Program/ Intervention**  - Not reported | **Barriers**  - Fairly high attrition rate (more than 70%)  **Facilitators**  - Increased awareness of increased vulnerability of psychosocial problems including a trauma history and behavioural health diagnoses  **Gaps**  - Not reported |
| Boglo et al., 2023 | UK | Cohort Study  To explore whether the  BIL intervention would result in the men who completed their  intervention demonstrating improved anxiety and depression | N = 50 men  Mean age [Years]: 40.77 (SD = 9.05)  Ethnicity  - White: 92%  - Black: 6%  - Asian: 2%  Years of formal education: "majority of participants had less than 10 years of formal education" | **Screening**  - BISI  - Mean TBI severity (using TBI Severity Index): 3.4 (SD = 1.53; severe)  - Mean number of blows to the head: 2.72 (SD = 1.46) | **Intersection**  - In prison  - On probation | **Intervention**  - The Brain Injury Linkworker Service – Linkworkers advocate for men by explaining consequences of brain injury and how it may affect cognitive abilities, behaviour, and emotional regulation  - Liaise with family and friend to provide information on how to support the individual when released  - "Through the gate" sessions for prison leavers and residents with their probation officers in the community  - Phase I – Identifying history and consequences  of TBI  - Phase II – Intervention in custody, tailored  to individual needs, person-centred  - Phase III – Follow-up after release  **Rehabilitation Team**  - Linkworkers (psychology graduates, clinical psychologist)  **Outcomes**  - Significant decrease in the scores reported on the GAD-7 and reduction in anxiety levels was observed in 80% of participants  - Decrease in self-reported depression on the PHQ-9  **Funding of Rehabilitation Program/Intervention**  - Not reported | **Barriers**  - Individuals were frequently released or relocated with little to no warning making it hard for the intervention to be completed  **Facilitators**  - Not reported  **Gaps**  - Does not explore experience of mental health and TBI in men from Black, Asian, and Minoritized Ethnic background |
| Trexler & Parrott, 2022 | USA | Non-randomized controlled study  To explore the extent to which resource facilitation (RF) may decrease  recidivism among those individuals with TBI | RF (N = 8)  Mean age [Years]: 39.13 (SD = 13.20)  Mean years of education: 11.88 (SD = 1.55)  Sex [Female]: 0%  Race  - White: 50%  - Black: 37.5%  - Other: 12.5%  Employed at time of arrest: 25%  Modified RF (N = 23)  Mean age [Years]: 40.43 (SD = 11.98)  Mean years of education: 10.57 (SD = 2.11)  Sex [Female]: 0%  Race  - White: 43.48%  - Black: 56.52%  - Other: 0%  Employed at time of arrest: 43.48% | **RF**  Screening  - OSU-TBI-ID  Mean age at first injury: 22.14 (SD = 17.99)  Injury severity  - Moderate: 62.5%  - Severe: 25%  **Modified RF**  Screening  - OSU Screening  Mean age at first injury: 23.27 (SD = 10.89)  Injury severity  - Moderate: 69.57%  - Severe: 26.09% | **Intersection**  - Parole/ community corrections program (administered by the Indiana Department of Corrections) | **Intervention: RF**  Comprehensive Evaluation and Education  - 8-hour evaluation of cognitive and neurobehavioural functions, substance abuse, family and social support, level of disability, pain, mobility, personality and emotional functioning, vocational preferences and barriers  - Individual community-based RF contact every 2 weeks on average for 12 months: facilitating resource acquisition, providing education to client, family, and providers, ongoing monitoring of success of resources, modification to plan as needed, collaboration and integration of treatment plans  - "Local support network community assessment"  - Monthly RF team case conference and report  documentation  - Vocational placement services for 90-day vocational stabilization  **Intervention: Modified RF**  Abbreviated Evaluation and Education  - Evaluation of cognitive/ neurocognitive functions, substance abuse, level of disability  - RF team case conference  - TBI wallet cards containing information about their injury and summary results of the evaluation were given to each client  - Person with TBI and family education about TBI  and its effects  - Navigation to multiple community services and supports (e.g., housing, medical services, brain injury  services, support groups, others) and management-coordination of services for comorbidities and co-occurring conditions (e.g., mental health, substance abuse)  - TBI Education and Life Skills Groups weekly  Rehabilitation Team  - Case managers  - TBI screening done by Master's level psychologist  **Outcomes**  - Fewer rearrests and incarcerations than those who did not receive RF (21% reduction in recidivism for RF group)  **Funding of Rehabilitation Program/ Intervention**  - US Department of Health and Human Services  - Health Resources and Services Administration  - Maternal and Child Health Bureau | **Barriers**  - Systematic disconnect between health and rehabilitation, criminal justice, and vocational and employment systems  **Facilitators**  - Not reported  **Gaps**  - Not reported |
| **BISI:** Brain Injury Screening Index; **CHAT:** Comprehensive Health Assessment Tool; **DUI:** Driving Under the Influence; **GAD-7:** Generalized Anxiety Disorder- 7; **HELPS**: Hit Head/ Been Hit on the Head, Emergency room, Loss of consciousness, Problems in daily life, Significant Sickness; **M2PI:** Mayo-Portland Adaptability Inventory- 4 Participation Index; **MPAI-4:** Mayo-Portland Adaptability Inventory- 4; **NRP:** Neurobehavioural Resource Project; **PHQ-9:** Patient Health Questionnaire- 9; **RF:** Resource Facilitation | | | | | | | |
